# Supplementary material for: Pathways Related to NLRP3 Inflammasome Activation Induced by Gold Nanorods
Source: Int J Mol Sci. 2022 May 20;23(10):5763. doi: 10.3390/ijms23105763 (PMC9145314; doi:10.3390/ijms23105763)
Supplement: Supplementary file 1 [file ijms-23-05763-s001.zip › ijms-1505031-supplementary.pdf]

International Journal of
*Molecular Sciences*

MDPI

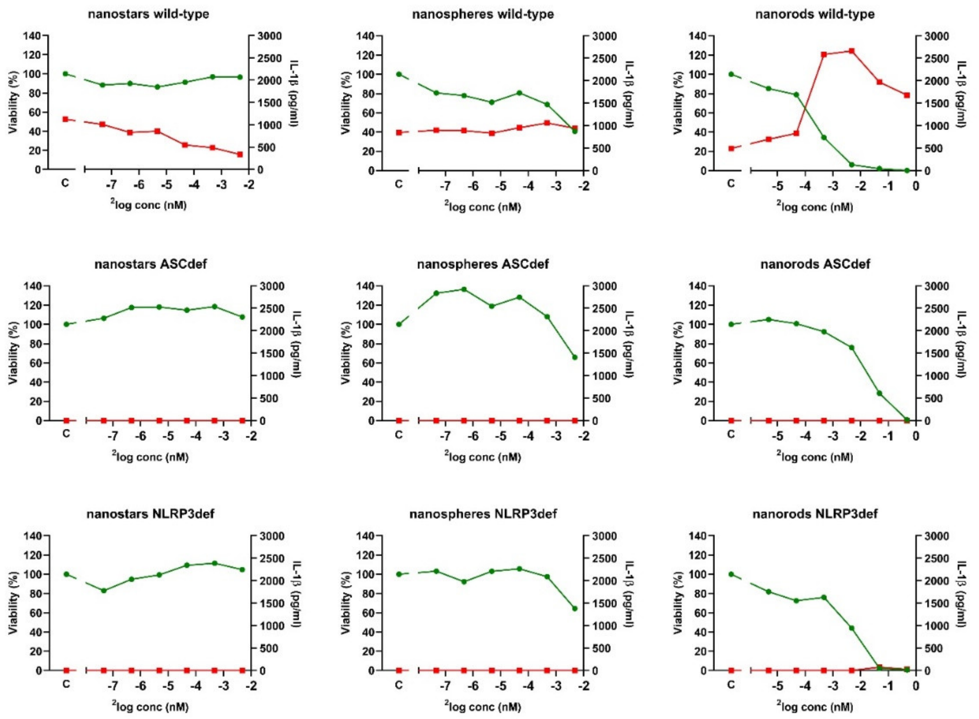


**Figure S1.** Exposure effects of gold nanostars (GNP-1), nanospheres (GNP-4), and nanorods (GNP-6) on cell viability (green) and IL-1β production (red) of cells derived from wildtype, ASC-deficient, and NLRP3-deficient THP-1 cells. Cells were PMA-stimulated for 3 h, allowed to rest for 24 h, and exposed for 48 h, after which, cell viability and cytokine production were measured. Nanoparticle concentration is expressed as ²log (nM). Viability: percentage of untreated controls. IL-1β: concentration in pg/mL. Representative result of N = 3 independent experiments. C: untreated control.
